# 

**Published:** 1890-02

**Authors:** 


### CONTENTS.

ORIGINAL COMMUNICATIONS—

THE IMPROVED CÆSAREAN SECTION. By W. D. Haggard, M. D, Nashville, Tenn ........ 715

IRITIS. By T. Hilliard Wood, M. D., Nashville, Tenn ........ 722

HAVE WE AN INFALLIBLE TREATMENT FOR DIPHTHERIA. By F. E. Waxham, M. D., Chicago, Ill ........ 728

CONCERNING CAT-GUT INFECTION. Translation from Centralblatt für Chirurgie ........ 732

SOCIETY REPORTS—

PHILADELPHIA OBSTETRICAL SOCIETY ........ 734

ATLANTA SOCIETY OF MEDICINE ........ 767

CORRESPONDENCE—

OUR NEW YORK LETTER ........ 772

BOOK REVIEWS—

ATLAS OF VENEREAL AND SKIN DISEASES. By Prince A. Morrow, A. M., M. D ........ 777

EDITORIAL—

THE HOSPITAL MOVEMENT ........ 780

"LA GRIPPE" ........ 781

ITEMS ........ 782

ERRATA ........ 782

